# Convolutional neural network-based kidney volume estimation from low-dose unenhanced computed tomography scans

**DOI:** 10.1186/s12880-023-01142-y

**Published:** 2023-11-15

**Authors:** Lukas Müller, Dativa Tibyampansha, Peter Mildenberger, Torsten Panholzer, Florian Jungmann, Moritz C. Halfmann

**Affiliations:** 1 Department of Diagnostic and Interventional Radiology, University Medical Center of the Johannes Gutenberg University Mainz, Langenbeckst, 1, 55131 Mainz, Germany; 2 Institute of Medical Biostatistics, Epidemiology and Informatics, University Medical Center of the Johannes Gutenberg University Mainz, Obere Zahlbacher Str. 69, 55131 Mainz, Germany

**Keywords:** Artificial intelligence, Convolutional neural networks, Segmentation, Kidney volume estimation, Low-dose CT

## Abstract

**Purpose:**

Kidney volume is important in the management of renal diseases. Unfortunately, the currently available, semi-automated kidney volume determination is time-consuming and prone to errors. Recent advances in its automation are promising but mostly require contrast-enhanced computed tomography (CT) scans. This study aimed at establishing an automated estimation of kidney volume in non-contrast, low-dose CT scans of patients with suspected urolithiasis.

**Methods:**

The kidney segmentation process was automated with 2D Convolutional Neural Network (CNN) models trained on manually segmented 2D transverse images extracted from low-dose, unenhanced CT scans of 210 patients. The models’ segmentation accuracy was assessed using Dice Similarity Coefficient (DSC), for the overlap with manually-generated masks on a set of images not used in the training. Next, the models were applied to 22 previously unseen cases to segment kidney regions. The volume of each kidney was calculated from the product of voxel number and their volume in each segmented mask. Kidney volume results were then validated against results semi-automatically obtained by radiologists.

**Results:**

The CNN-enabled kidney volume estimation took a mean of 32 s for both kidneys in a CT scan with an average of 1026 slices. The DSC was 0.91 and 0.86 and for left and right kidneys, respectively. Inter-rater variability had consistencies of ICC = 0.89 (right), 0.92 (left), and absolute agreements of ICC = 0.89 (right), 0.93 (left) between the CNN-enabled and semi-automated volume estimations.

**Conclusion:**

In our work, we demonstrated that CNN-enabled kidney volume estimation is feasible and highly reproducible in low-dose, non-enhanced CT scans. Automatic segmentation can thereby quantitatively enhance radiological reports.

## Key points

• CNN-enabled kidney volume estimation is highly reproducible in low-dose, non-enhanced CT scans.

• CNN-enabled volume estimation required a fraction of time required by semi-automatic segmentations.

• Development of CNN-based models can be facilitated by pre-existing structured reporting environments.

## Introduction

Kidney volume is an important parameter in the diagnosis, therapy and long-term management of renal diseases [1]. Patients with a predisposition for urolithiasis are prone for recurrences and therefore undergo repetitive CT scans. While obstructive urolithiasis with concomitant infection can lead to swelling of the renal parenchyma in the acute phase, chronic kidney disease as well as recurrent infections commonly lead to a decrease in renal volume. Therefore, quantification of renal volume would yield additional clinical information. However, it is rarely performed because manual or semi-automated estimation of kidney volumes is cumbersome, requires additional software and disrupts reporting workflows. With the recent advances in Artificial Intelligence (AI), assessment of standardized image data can be economized and automated. AI can assist radiologists in image analysis by extracting visual information that is invisible to, or hard to detect by, the human eye. In recent years, several AI applications to medical image analysis such as organ and lesion segmentation, classification, localization, detection, and registration have been presented [2–5]. In fact, AI has been demonstrated by some researchers to achieve higher performance than radiologists, for example for breast cancer detection in mammography [6].

Convolutional Neural Networks (CNNs) are currently the most adopted class of AI deep learning algorithms in image analysis. This is enabled mainly by their ability to extract features of an image and reduce its dimensionality, while preserving local spatial relations [7]. This, in turn, reduces the number of parameters the algorithm has to compute, hence increasing speed and efficiency. Despite their major drawback of lack of transparency (“black box”), CNNs and Neural Networks in general have been demonstrated to provide reliable results.

For the kidneys, however, the volume estimation is still a challenge, because their position varies and they are surrounded by tissues of similar density [8]. Due to poor acoustic windows and dependence on the observers’ experience, this problem can be even more accentuated in ultrasound sonography, which is typically performed prior to confirmatory the CT. Previous segmentation algorithms have overcome these problems for contrast-enhanced CT scans, which yield higher contrasts between the perirenal space and the kidney parenchyma. Some other have successfully exploited high attenuation differences, such as those observed between fluid-filled cysts and kidney parenchyma. Multiple deep learning algorithms using CNNs have been proposed to segment kidney volumes and cysts for follow-up scans in autosomal dominant polycystic kidney disease (ADPKD) [8–11]. Keshwani et al., applied a 3D U-net-based CNN for kidney segmentation on (mostly non-contrast enhanced) CT scans of patients diagnosed with ADPKD [12]. Chantaduly et al., applied a 2D U-Net-based CNN segmentation for quantification of kidney fibrosis using regular-dose CT scans (79% without intravenous contrast) of patients for kidney biopsy [13]. Additionally, various approaches using either a 3D U-Net-based ensemble CNN [14] or a segmentation-free CNN [15] were developed for patients diagnosed with kidney tumors. A ResNet-based CNN was trained for kidney segmentation on arterial phase contrast-enhanced CT images of kidney cancer patients [16]. However, none of these methods have been applied to low-dose unenhanced scans, which are routinely acquired in patients with suspected urolithiasis [17]. In addition to the absence of contrast, low-dose unenhanced scans also suffer from a greater amount of image noise due to the lower radiation doses applied. Despite multiple commercially available segmentation tools on the market, none can process low-dose unenhanced scans.

The aim of this study was therefore to establish an automatic evaluation of kidney volume in non-contrast, low-dose CT scans of patients with suspected urolithiasis. To this end, we trained our own 2D CNN model to automatically segment kidney regions used to calculate the kidney volume. In the following, we report a successful application of the 2D CNN, based on ‘U-Net’ architecture [18] for kidney segmentation and its application to kidney volume estimation.

## Material and methods

### Study population and image acquisition

**Fig. 1:**
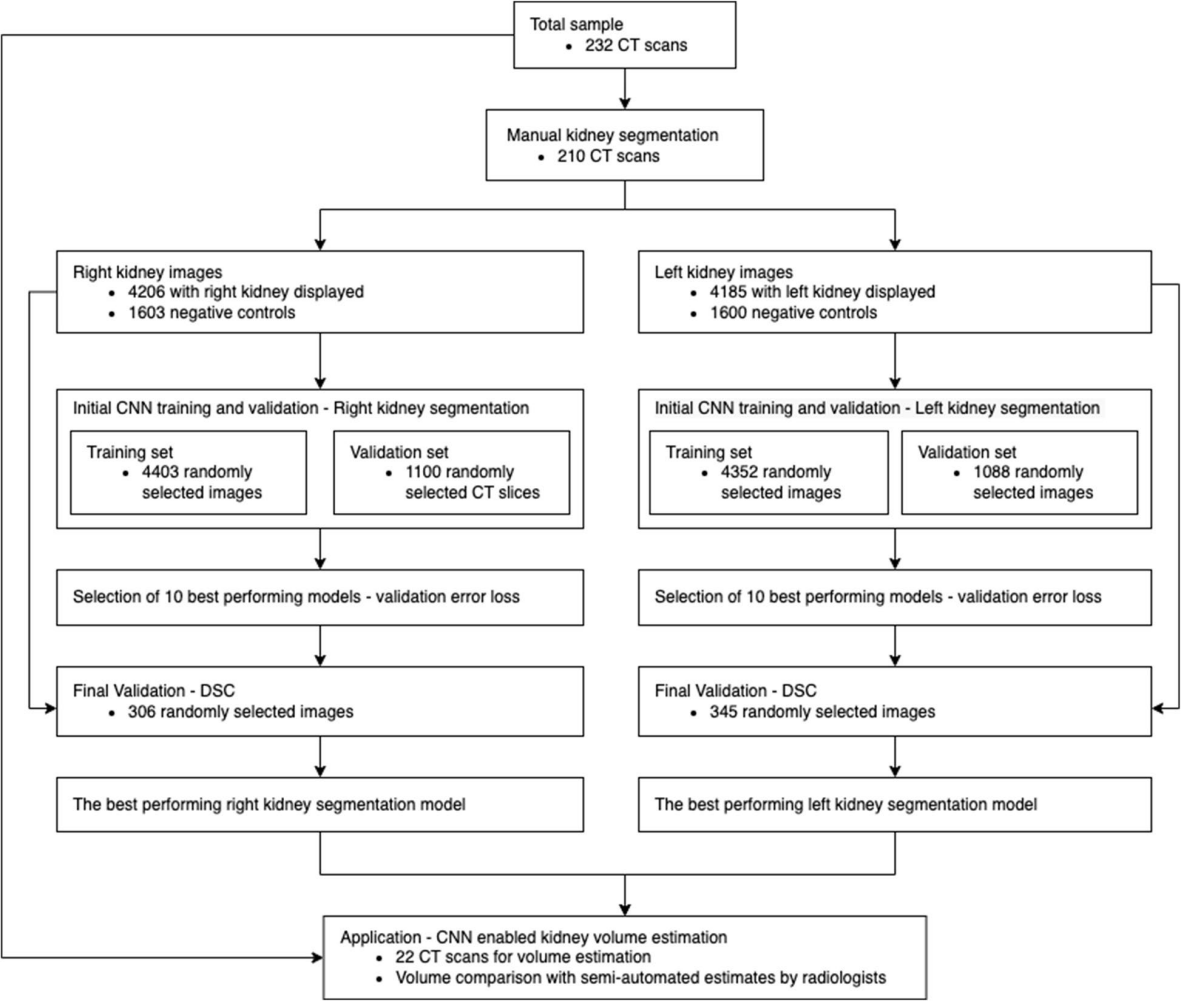
A schematic summary of the study

The study population (Fig. 1) consisted of 232 retrospectively identified consecutive unenhanced low dose CT scans of the retroperitoneum obtained between 01/2019 and 12/2019 at the University Medical Center of the Johannes Gutenberg University Mainz, a tertiary hospital. All scans were acquired with a 64 slice (Brilliance 64, Philips Healthcare) or 256 slice (iCT 256, Philips Healthcare) CT scanner, with an acquisition slice thickness of 0.625 mm, a body convolution reconstruction kernel, reconstruction thicknesses 1 and 3 mm, 120 kVp tube voltage and body weight adapted fixed tube current. Based on institutional standard operating procedures for the acquisition of clinical images, the image quality of all CT scans was verified by a radiologist after acquisition of the clinical scan. If images were not of diagnostic quality (e.g. due to breathinig artifacts or too much noise in the low-dose scans), the scan was repeated. Due to the retrospective nature of this study, this means all included scans were already screened no additional image quality assessment was performed for the purpose of the study.

This retrospective, single-site, cohort study did not need professional legal guidance by the Institutional Review Board or informed consent of patients according to the state hospital law. All analyzed patient data was fully de-identified. Twenty percent of CT scans were from patients with no apparent renal disease, the remaining 80% were from patients diagnosed with urolithiasis. Gender and age were reported for only 222(96%) of the patients. Of these 34% were female and 66% were male. The age in years for these patients ranged between 15 and 91, with an average age of 50, median of 49 and interquartile range of 23.25.

### Image extraction and manual kidney segmentation

Transverse CT slices were extracted from the training set of 210 CT scans using the Digital Imaging and Communications in Medicine (DICOM) viewer Sectra Workstation (IDS7, Version 24.1, Sectra AB, Sweden) [19]. Slices covering the entire vertical length of both kidneys were extracted at intervals of 10 mm. Side-matching slices taken randomly from the area above and below the kidneys, were added as negative controls. The left and right kidneys were then manually segmented by two radiology students under the guidance of an expert, using the Ratsnake annotation tool (v1.4, is-innovation.eu, Lamia, Greece) [20]. Thus, the segmented masks were used to generate training and validation datasets for the CNN. Incomplete data was excluded from the datasets i.e. images displaying kidney for which no mask was generated. The remaining 22 unseen CT scans were used as an application dataset for the final comparison between the CNN-enabled and semi-automated kidney volume estimation (Fig. 1).

### Image adjustment

To improve applicability to a general population (e.g. patients with a single kidney) and address anatomical variance in kidney positioning, all images were split vertically into two parts with an overlap of 5% of the width at the image center. This resulted in two separate datasets, one for the right kidney and one for the left kidney. The final sets contained:

- Right kidney dataset: 5809 transverse CT slices (4206 displaying right kidney, 1603 negative controls).
- Left kidney dataset: 5785 transverse CT slices (4185 displaying left kidney, 1600 negative controls),

### CNN architecture

**Fig. 2:**
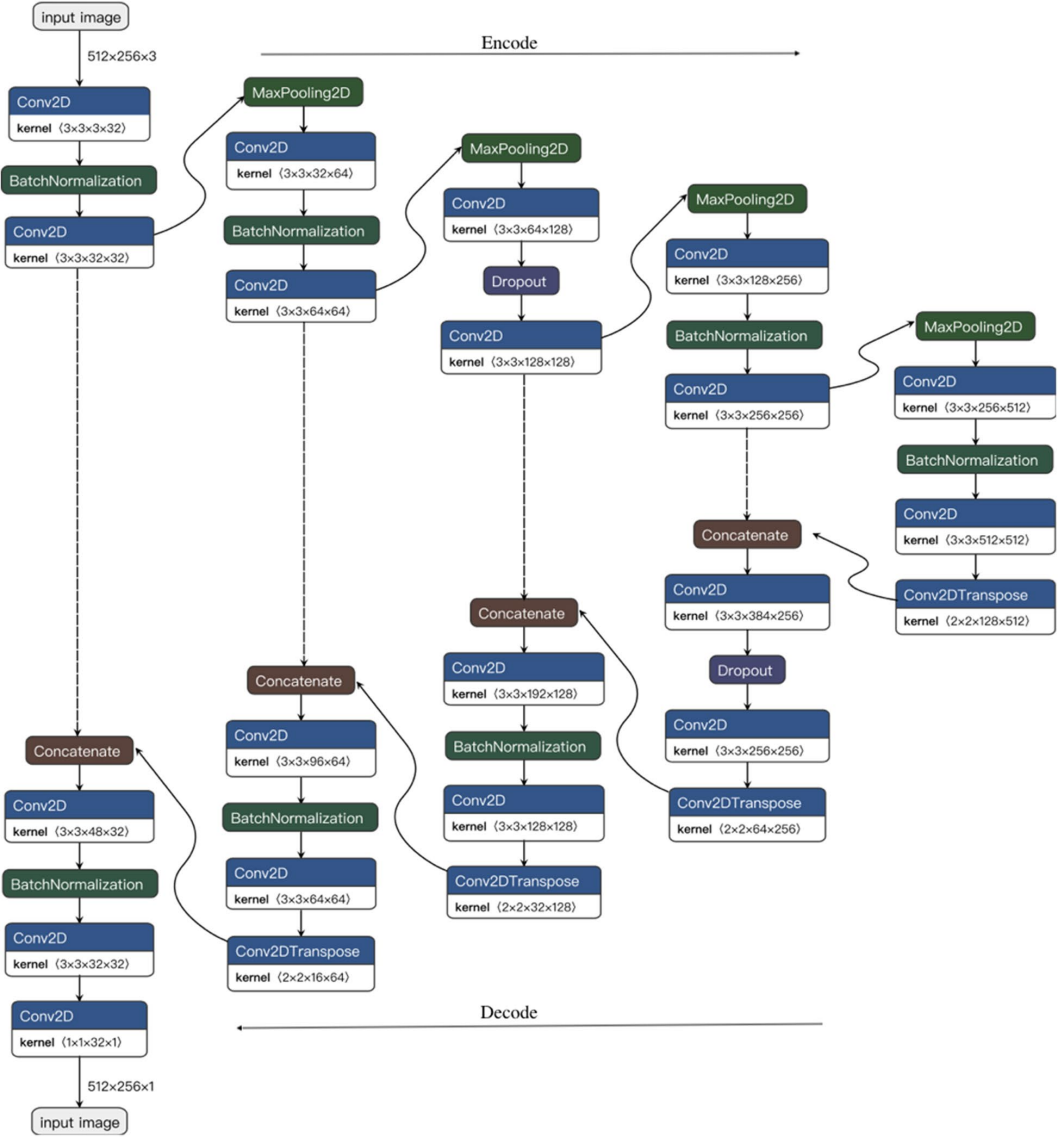
The architecture and operations carried out by the U-Net CNN. Kernel refers to the shape of a tensor with the following dimensions: height of the kernel, width of the kernel, number of input channels, number of output channels

We adopted the 2D U-Net CNN architecture [18] for training our kidney segmentation models. U-Net is a modified version of the fully connected neural network that has been used for medical image analysis across different organs and has previously shown high segmentation and localization accuracy. U-Net is commended for its ability to work well on smaller image datasets and its ability to train fast. The architecture and operations of the U-Net based CNN as implemented for our model training, are explained in the subsequent paragraphs and illustrated in Fig. 2.

The first half of the architecture is the encoder, which extracts feature representations from the input image. The encoder consisted of 10 convolutional layers. Each convolutional layer was alternatively followed by either a max-pooling operation or a batch normalisation operation. Only the fifth layer was followed by a dropout operation. This sequence of convolution and max-pooling operations results in a gradual increase in the number of extracted features, while simultaneously reducing the location characteristics.

The second half of the architecture is the decoder, which recovers and restores the original image resolution of the extracted features. The features learned by the encoder are projected onto the corresponding higher resolution plane from the contraction path to obtain a high-resolution segmented image. The decoder consisted of four transpose convolution layers. Each transpose convolution layer was followed by a concatenation operation to concatenate the output of the transpose layer with that of the corresponding layer in the decoding phase, and then a convolution operation. The first convolution layer was followed by a drop-out operation and every other second convolution was followed by a batch normalization process, with exception of the final convolutional layer.

Altogether, the CNN architecture implemented for our model training (Fig. 2) consisted of 18 convolutional layers, each with a 3 × 3 kernel of 2 two strides, and a Rectified Linear Unit (ReLU) activation function. The number of feature maps for the first two convolutional layers was 32, and was increased by a factor of 2 for each of the next two convolutions and reduced by a factor of 2 for in the deconvolution process. There were also four 2 × 2 max-pooling layers with strides of 2, and four transpose convolution layers with 2 × 2 kernels. The number of feature maps in each transpose convolution layer was the same as that of the preceding convolutional layer. The last convolutional layer had a 1 × 1 kernel and a sigmoid activation function. As CNNs are prone to overfitting and slow convergence, two dropout layers and seven batch normalization operations were introduced to improve the generalization of the CNN model [21, 22].

### CNN implementation and training

The 2D CNN was implemented with Tensorflow (v2.1.0, Google LLC, Mountain View, USA), Keras backend (v2.3.1, Francois Chollet, Google LLC, Mountain View, USA), and Python (v3.6.9, Python Software Foundation, Wilmington, Delaware, United States). Each kidney side was trained separately from scratch, resulting in two distinct sets of CNN models. The initial training used 5503 and 5440 2D images of the right and left kidneys respectively. Eighty percent of these images were used for the initial training and 20% for validation.

The training of a CNN is critically affected by the applied hyper-parameters, manual optimization of which is time-consuming. We applied a genetic algorithm to automatically search and optimize the learning rate, dropout rate, starting number of feature maps, and batch size hyperparameters [23, 24]. Ten sets of four randomly selected hyperparameters were generated, with each set consisting one of each of the hyperparameters listed above. These 10 sets served as the population of the genetic algorithm, each parameter set as an individual within the population, and the parameters in a set as the genetic make-up of each individual. The genetic algorithm was trained on subsets of 25% randomly selected images from each kidney side. A new generation resulted from the half-fittest parameter sets and their crossover with single-point mating. All parameters in a set, except those of the fittest parameter set, were subjected to 20% probability of Gaussian mutation i.e. the mutant parameter was replaced by a value generated according to the Gaussian distribution around the original parameter value. The optimal parameters obtained after 25 generations were: learning rate 0.15, batch size 12, feature maps 32 and dropout rate 0.1. Thus, optimized hyper-parameters were adopted for training our CNN models.

In each epoch, each image was subject to 50% probability of augmentation. An image could undergo only one type of augmentation i.e. optical distortion, multiplicative noise or Gaussian blur. Image augmentation was implemented using Albumentations library (v0.5.2, https://albumentations.ai) [25]. The CNN input images were resized to 512 × 256 pixels. The pixel intensity values were normalized to a range of 0–1.

The CNN models were trained from scratch on a workstation with two NVIDIA Corporation GP100GL [Tesla P100 PCIe 16 GB] GPUs, taking approximately 9 h to make one round of training. For optimization, we used the Adam optimization function and binary cross-entropy for the loss function. Early stopping was applied after 30 consecutive epochs of no improvement in the validation error loss. Several rounds of training were carried out, resulting in multiple trained models for either kidney side.

### Evaluation of the resulting CNN models

#### CNN model evaluation

The validation error loss metric, which measures how well the trained CNN models fit to the training validation data sets, was used through select the best 10 performing models for each kidney side. As a final validation, the overlaps between the CNN model-generated masks were compared against the manually-generated masks using the statistical metric DSC [26]. The CNN models from each kidney side that generated the highest DSC were selected for application for kidney volume estimation.

### Volume estimations and analyses

#### Semi-automated volume estimation

The semi-automated kidney volume estimation was carried out by two experienced radiologists on 22 CT scans using dedicated freeware annotation software (LifeX v7.2.8, C. Nioche, Inserm, www.lifexsoft.org). The number of slices per CT ranged between 750 to 1401 slices. On average each CT scan had 1026 slices. These CT scans were used neither in the training, nor in the aforementioned validation of the CNN models.

To estimate the volume, the same radiologists manually outlined the kidney area and the software estimated the area of the outlined segment. This was done for images corresponding to the kidney region and positioned at 3 mm apart. The total kidney volume was obtained by summing up the products of the segmentation areas with the distance between subsequent image slices.

#### CNN-enabled volume estimation

The volume calculation followed similar steps as in the aforementioned semi-automated volume estimation method. The CNN models were used to segment the kidneys areas. The only difference was that the CNN-enabled kidney volume estimation method utilized all the available image slices in the CT scan (1 mm apart), i.e. approximately 3 times more images than the semi-automated volume estimation method. The same dataset of 22 CT used in the semi-automated volume estimation method were also used here. All CT slices had 512*512-pixel dimensions. The slices were cropped to dimensions of 512*256 pixels for each kidney side and fed into the CNN models for automatic kidney segmentation. The voxel volumes in each segmented mask were calculated from the x and y pixel spacing and spacing between slices provided in the DICOM headers of every slice. The total kidney volume was then calculated from the summation of product of the voxels and their volume in each mask (Eq. 1).

1 $$\mathrm{Volume}=\sum_{i-1}^{n}{x}_{1}\times {y}_{1}\times {z}_{i}\times {No. pixels}_{i}$$

*Where i* = *CT slice, n* = *number of slices with kidney masks, x*_*i*_ = *x dimension pixel spacing, y*_*i*_ = *y dimension pixel spacing and z* = *distance between subsequent slices. No. Pixels represents the number of pixels in the segmented mask.*

**Fig. 3:**
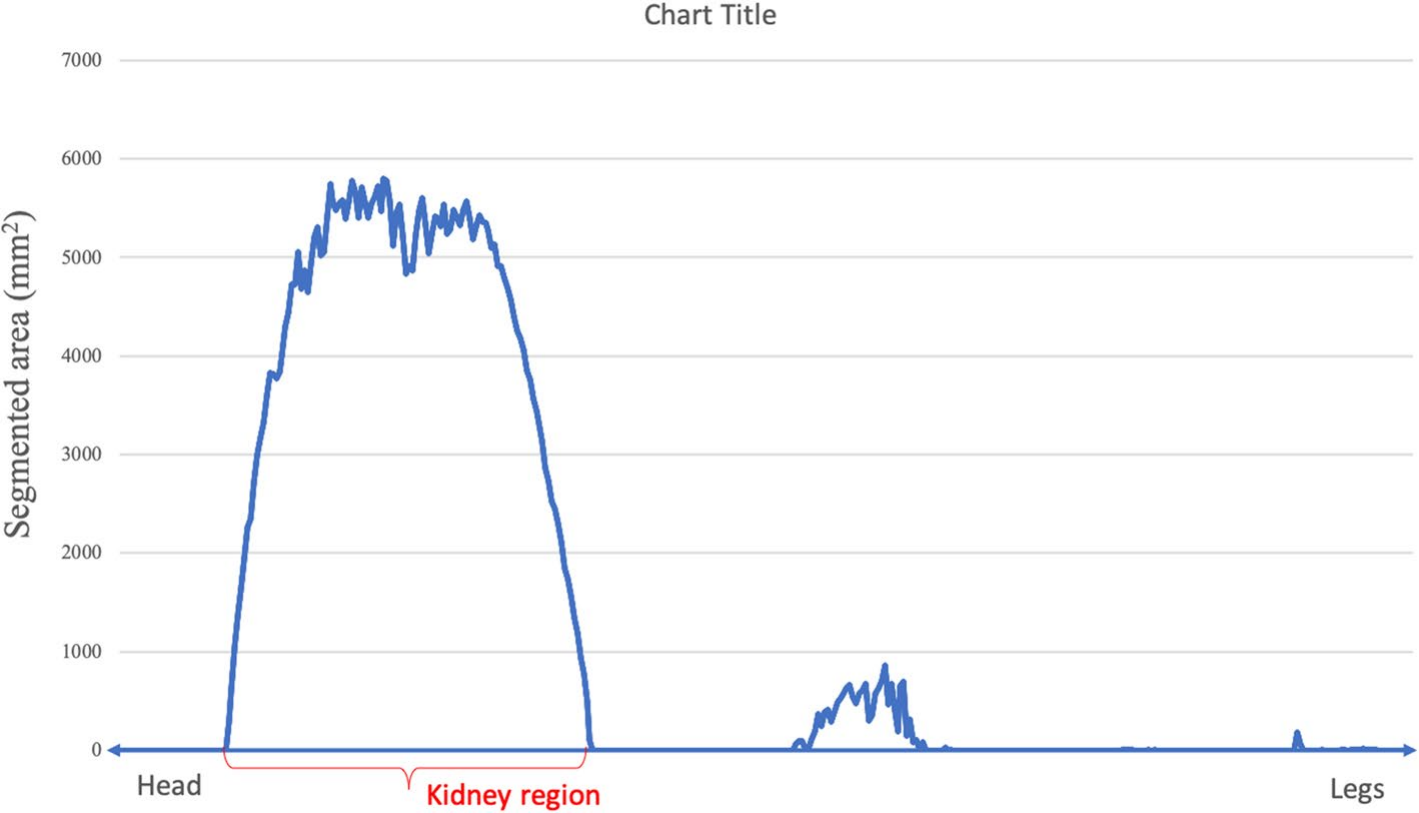
An example of pixel distribution segment-by-segment. The uninterrupted series of segments corresponding to the left kidney is marked in red

There were a few occurrences where the CNN models wrongfully segmented structures in some slices that clearly contained no kidney. To exclude such erroneous masks from kidney volume calculation, the kidney region was identified by assessing the pixel distribution sorted according to the table positions (Fig. 3). The largest uninterrupted series of images containing masks with large pixel numbers was considered to be the kidney region. Further, a minimum pixel count was set to 200, since the CNN models in most patient cases failed to segment the smallest kidney areas i.e. the start and end of the kidney.

#### Statistical analysis of kidney volume

All statistical analyses were performed using dedicated software (R v4.2.0, R Core Team, www.r-project.org). Agreement between CNN-enabled and semi-automated volume estimations was evaluated using Bland-Altmann plots. The limits of agreement were calculated by using the mean and the standard deviations of the differences between two methods.

Agreement of repetitive measures within the methods, as well as between CNN-enabled and semi-automated volume estimates, were evaluated by means of intraclass correlations coefficients (ICC). The ICC estimates and their 95% confidence intervals were calculated based on a two-way mixed-effects model—single rater absolute agreement and consistency. Levels of agreement were defined as followed: poor, ICC < 0.5; moderate, ICC = 0.5–0.75; good, ICC = 0.76–0.9; excellent, ICC > 0.9 [27]. Additionally, the performance of the models was evaluated using mean absolute error (MAE), and Median Absolute Percentage Error (MdAPE).

## Results

### CNN-enabled kidney segmentation

**Fig. 4:**
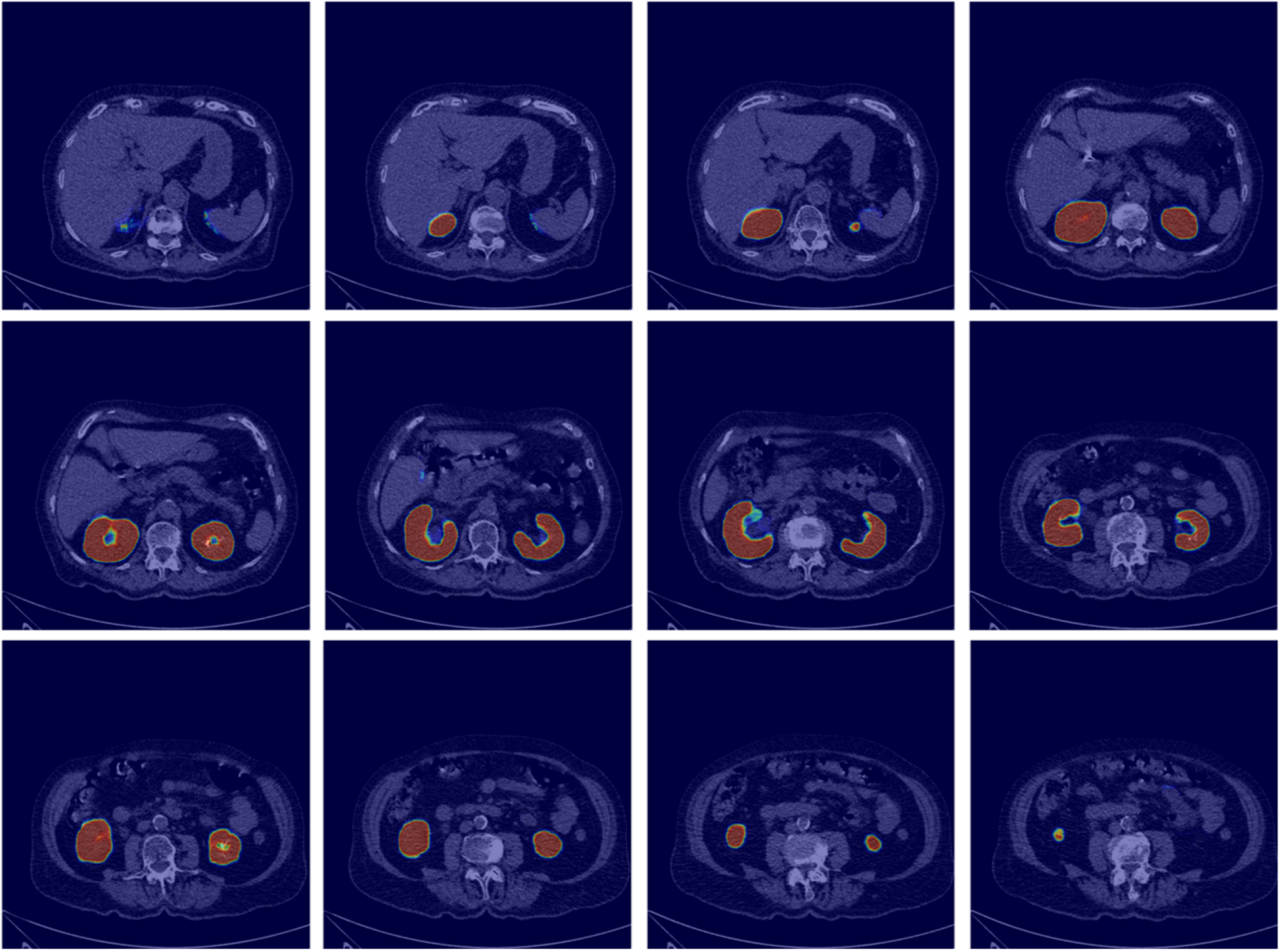
Grad-cam visualization of the last layer of the model on randomly selected test images

**Fig. 5:**
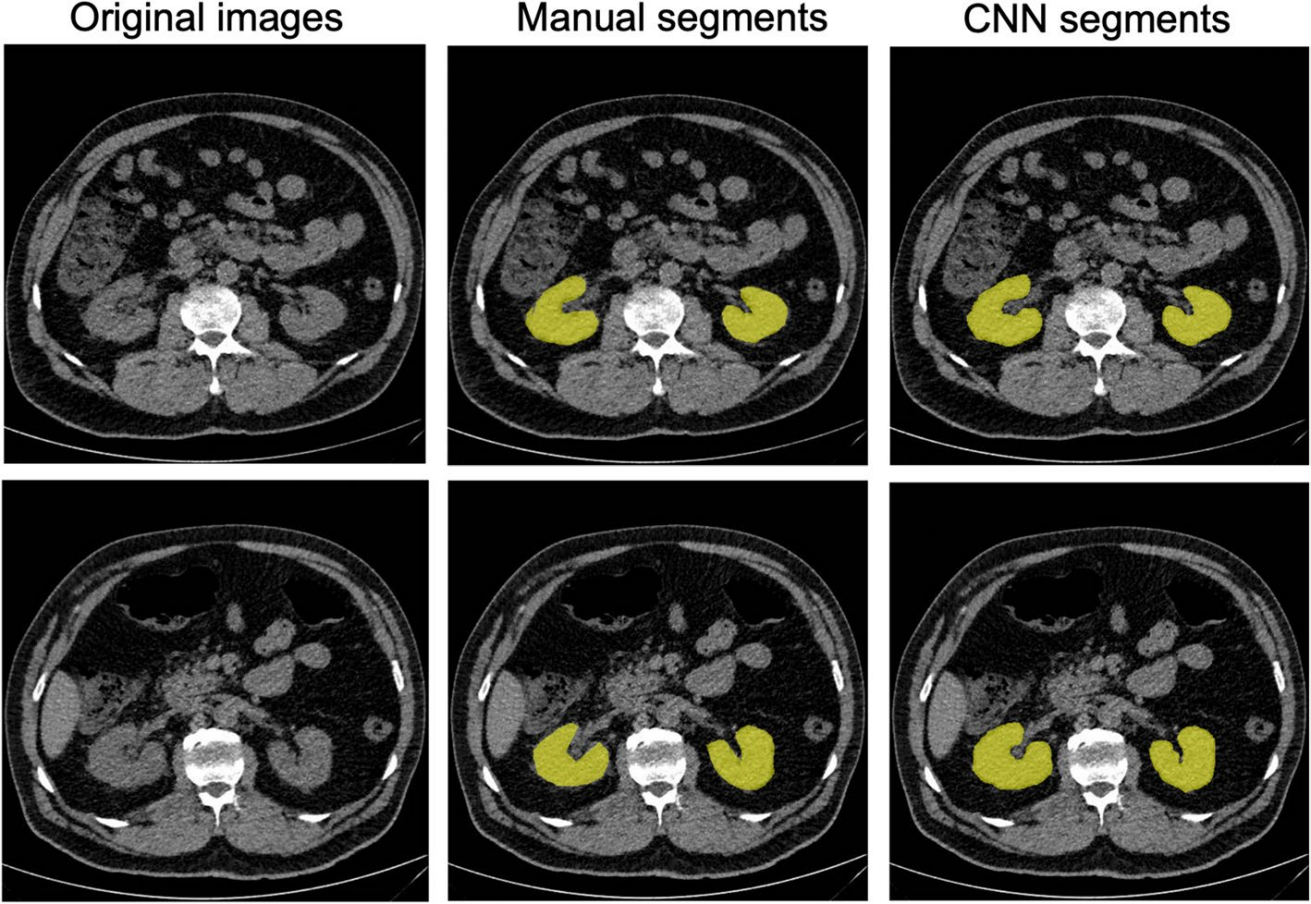
Visualization of randomly selected manual segments by radiology students versus CNN-derived segments from the final validation dataset

The CNN segmentation models were subject to DSC analysis on a subset of 5.5/5.9% of the right and left kidney images (306/345 images, respectively) which had not been used during the training processes. The models that yielded the highest DSC scores for either kidney side were applied for volume estimation. The DSC scores for the selected right and left kidneys models were 0.91 and 0.86, respectively. Figure 4 shows the heatmaps generated by Grad-CAM for random test images. It is clear from Fig. 4 that the models looked exactly where we wanted them to look, i.e. at the location of the kidneys. A randomly chosen sample of the masks produced by the CNN segmentation models with their comparisons to those generated manually is shown in Fig. 5. The results of applying these CNN models to volume estimation are shown in one of the subsequent sections.

### Volume estimations and comparisons

#### Semi-automated volume estimates

**Fig. 6:**
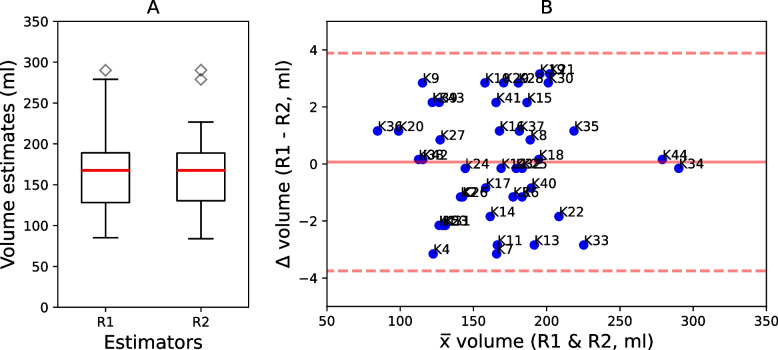
**A** Boxplots for the comparison of semi-automatic kidney segmentation results between readers. **B** Bland-Altmann Plots comparing the semi-automatic kidney segmentation. K represents a kidney

Two radiologists (referred to as R1 and R2) used a semi-automated method to estimate volume of 44 kidneys. On average it took the radiologists 22.09 min to estimate volume of two kidneys from one patient. The volume estimates ranged between 84 and 290 ml, with a mean of 166 ml and standard deviation of 41 ml (Fig. 6A). The MAE and MdAPE were 1.6 and 1.15% respectively. In the Bland–Altman Plot (Fig. 6B), the difference between R1 and R2 is plotted against their mean. On average, R2 estimated 0.1 ml higher than R1 (mean: 0.1 ml; 95% CI = -3.9 ml; 3.9 ml). The inter-rater variability showed an excellent consistency of ICC $\ge$ 0.99 and an excellent absolute agreement of ICC $\ge$ 0.99 among the radiologists.

#### CNN-enabled volume estimates

The selected CNN models were applied to automatically segment the kidney regions in all the 22 test CT scans. The voxels in the segmented masks were used to estimate the total kidney volume for each kidney respectively. As distinct CNN models were generated for either kidney side, the analyses are described separately.

#### Right kidney

**Fig. 7:**
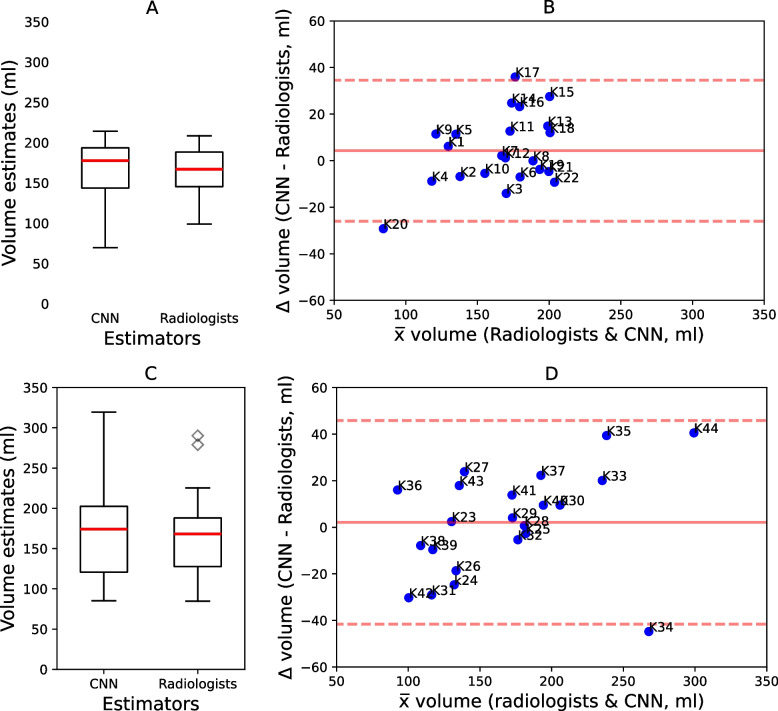
Comparison of CNN with semi-automatic volume estimates. K represents a kidney. **A** and **B** are for volumes estimates of the right kidneys. **C** and **D** are for volumes estimates of the left kidneys

The right CNN model was applied for volume estimation of the 22 right kidneys. Repeating the volume estimation process 20 times on each kidney generated identical volume estimates. On average, it took the CNN-enabled method 32 s to estimate volume for one kidney. The absolute CCN-enabled volume estimates ranged between 70 and 214 ml, with a mean of 168 ml and standard deviation of 35 ml. The average volume estimates by radiologists ranged between 99 ml - 208 ml, with a mean of 163 ml and standard deviation of 30 ml (Fig. 7A). The MAE and MdAPE were 12.4 and 6.5% respectively. Figure 7B, the difference between the two volume estimation methods is plotted against their mean. On average, the CNN-enabled method estimated volumes were 4 ml higher than radiologists (mean: - 4.3 ml; 95% CI = -26.0 ml; 34.5 ml). Assessment of inter-rater variability showed a good consistency of ICC = 0.89 and a good absolute agreement of ICC = 0.89 between the two methods.

#### Left kidney

The left CNN model was applied for volume estimation of the 22 left kidneys. Repeating the volume estimation process 20 times on each kidney generated identical volume estimates. On average it took the CNN-enabled method 31 s to estimate volume for one kidney. The absolute CNN-enabled volume estimates ranged between 85 and 319 ml, with a mean of 170 ml and standard deviation of 59 ml. The average volume estimates by radiologists ranged between 84 and 290 ml, with a mean of 168 ml and standard deviation of 51 ml (Fig. 7C). The MAE and MdAPE were 17.9 and 9.8% respectively. In Fig. 7D, the difference between the two volume estimation methods is plotted against their mean. On average, the CNN-enabled method estimated volumes 2 ml higher than the radiologists (mean: 2.1 ml; 95% CI = -41.57 ml;45.8 ml). Assessment of inter-rater variability showed an excellent consistency of ICC = 0.92 and an excellent absolute agreement of ICC = 0.93 between SE and RA volume estimates.

## Discussion

This study evaluated the application of CNN-enabled kidney segmentation to kidney volume estimation in low-dose, unenhanced CT scans of the retroperitoneum. The main findings were: 1) There was an excellent internal reproducibility within the CNNs for the separate estimation of left and right kidney volumes 2) Application and comparison to a previously unseen dataset revealed good agreement between CNNs and semi-automated measurements 3) there was a slight, clinically insignificant, overestimation of kidney volumes by the CNNs when compared to semi-automated measurements.

Only few previously developed approaches for automated kidney segmentation included unenhanced CT scans in their training data. For example, Keshwani et al. trained a 3D U-net-based FCN for kidney segmentation in patients diagnosed with ADPKD [12]. These patients typically have numerous fluid-filled cysts throughout their kidneys which lead to severe organ enlargement. While segmentation of these kidneys poses its own challenges such as liver cysts coexisting in close proximity [28], the results cannot be transferred to our study cohort and a model trained on ADPKD patients might not perform well in patients with suspected urolithiasis. This is due to the fact that heavily enlarged, cystic kidneys occupy almost the entire retroperitoneum, requiring the model to differentiate the borders of fluid-filled cysts from lumbar muscle tissue or the abdominal wall, rather than kidney parenchyma from the peri-renal fat. In addition, Keshwani et al. reported that their model was sensitive to image quality and only used regular-dose, unenhanced scans. In comparison, our study was based on low-dose scans typically showing greater amounts of image noise, which in turn leads to lower signal-to-noise ratios, making the differentiation between tissues harder. Low-dose represents a bulk scans used for diagnosing urolithiasis [17]. Similar to previous studies, we found that volumes of the left kidney tended to be larger than on the right side [29, 30]. The most likely explanation for this is the topographical proximity to the liver which takes up more space than the spleen on the left side. Another factor contributing to size differences might also be blood flow, as the left renal artery is typically shorter than the right one.

Manual kidney volume estimation can be cumbersome and time-consuming as it requires additional software and therefore disrupts a radiologists’ workflow in clinical routine. In agreement with previous reports, this study found that semi-automated kidney segmentation took approximately 22 min per patient [10–22]. This is especially relevant as this can far exceed the amount of time required to read the scan and write up the radiology report.

The development, training and validation of CNN for automated organ segmentation has its specific requirements. Primarily, the setup of a large and detailed enough databank is required and thus digital access barriers can hinder the development of new CNNs. As demonstrated in this case, the training can be facilitated by routinely feeding clinical scans in a structured reporting database, which can be automatically read according to indications and even structured reports. In doing so, the presented CNN-enabled method does not only deliver fast (less than 1 min) and reproducible kidney volumes estimates but could also be trained on pre-prepared data, significantly speeding up the development process.

Before productive use, the safety and performance requirements according to the EU Directive on Medical Devices (93/42/EEC) must additionally be tested [31]. Once implemented, the CNN-based model can then automatically feed its data into a structured reporting environment, as well as the local Picture Archiving and Communication System (PACS), and thereby economize data-driven radiological reports [32]. Using this feedback-loop has two beneficial effects: Firstly, daily clinical work of the radiologist can be accelerated while also enriching reports with more evidence based data [33]. Secondly, the gathered data is well-structured and easily accessible to be used for the further evaluation of the implemented model in regard to clinical implications of the segmentation data.

Despite including a large cohort of 232 CT scans, our study is limited due to cross-sectional single-center design and the relatively small sample size of the application set. In addition, although the scans were acquired on two different scanners to which the CNN was blinded, the generalizability of the segmentation model could be further improved with a more diverse scanner set. The segmentation model was trained and applied only to unenhanced CT scans. Therefore, it is unclear how it would perform in contrast-enhanced scans. As there is no gold standard of the kidney volume estimates, the CNN-enabled volume estimates were compared against the average estimates of the experts that participated in this study.

## Conclusions

We report the successful implementation of a 2D CNN-enabled kidney segmentation model trained on low-dose unenhanced CT scans of the retroperitoneum and its application to kidney volume estimation. We highly encourage the use of a structured database for the training and validation of the CNNs as well as the implementation of a feedback-loop back into the system to not only improve radiological reports, but also to enable comprehensive further evaluation of the CNNs clinical implications. Our next step is to use this data to conduct a longitudinal study to assess the relation of kidney volume change and renal function over time.

## Data Availability

The datasets used and/or analyzed during the current study are available from the corresponding author on reasonable request.

## Abbreviations

ADPKD: Autosomal Dominant Polycystic Kidney Disease
AI: Artificial Intelligence,
CNN: Convolutional Neural Networks
CT: Computed Tomography
DICOM: Digital Imaging and Communications in Medicine
DSC: Dice Similarity Coefficient
ICC: Intraclass Correlations Coefficients
PACS: Picture Archiving and Communication System
ReLU: Rectified Linear Unit

## References

[1] Grantham JJ, Torres VE (2016). The importance of total kidney volume in evaluating progression of polycystic kidney disease. Nat Rev Nephrol.

[2] Litjens G (2017). A survey on deep learning in medical image analysis. Med Image Anal.

[3] Fu Y, Lei Y, Wang T, Curran WJ, Liu T, Yang X (2020). Deep learning in medical image registration: a review. Phys Med Biol..

[4] Cai L, Gao J, Zhao D (2020). A review of the application of deep learning in medical image classification and segmentation. Ann Transl Med.

[5] Sahiner B (2019). Deep learning in medical imaging and radiation therapy. Med Phys.

[6] Rodriguez-Ruiz A (2019). Stand-alone artificial intelligence for breast cancer detection in mammography: comparison with 101 radiologists. JNCI J Natl Cancer Inst.

[7] LeCun Y, Kavukcuoglu K, Farabet C. Convolutional networks and applications in vision. In: Proceedings of 2010 IEEE international symposium on circuits and systems, IEEE; 2010. p. 253–256.

[8] Thong W, Kadoury S, Piché N, Pal CJ (2018). Convolutional networks for kidney segmentation in contrast-enhanced CT scans. Comput Methods Biomech Biomed Eng Imaging Vis.

[9] Jagtap JM (2022). Automated measurement of total kidney volume from 3D ultrasound images of patients affected by polycystic kidney disease and comparison to MR measurements. Abdom Radiol NY.

[10] Goel A (2022). Deployed deep learning kidney segmentation for polycystic kidney disease MRI. Radiol Artif Intell.

[11] Sharbatdaran A (2022). Deep learning automation of kidney, liver, and spleen segmentation for organ volume measurements in autosomal dominant polycystic kidney disease. Tomography.

[12] Keshwani D, Kitamura Y, Li Y. Computation of total kidney volume from CT images in autosomal dominant polycystic kidney disease using multi-task 3D convolutional neural networks. ArXiv180902268 Cs. 2018. Available: http://arxiv.org/abs/1809.02268. Accessed 9 Nov 2021.

[13] Chantaduly C, Troutt HR, Perez Reyes KA, Zuckerman JE, Chang PD, Lau WL (2021). Artificial Intelligence Assessment of Renal Scarring (AIRS Study). Kidney.

[14] Fatemeh Z, Nicola S, Satheesh K, Eranga U (2020). Ensemble U-net-based method for fully automated detection and segmentation of renal masses on computed tomography images. Med Phys.

[15] Hussain MA, Hamarneh G, Garbi R (2021). Cascaded regression neural nets for kidney localization and segmentation-free volume estimation. IEEE Trans Med Imaging.

[16] Hsiao CH, et al. Automatic kidney volume estimation system using transfer learning techniques. In: Barolli L, Woungang I, Enokido T, editors. Advanced information networking and applications, in lecture notes in networks and systems. Cham: Springer International Publishing; 2021. p. 370–381. 10.1007/978-3-030-75075-6_30.

[17] Weinrich JM (2018). Low-dose CT for evaluation of suspected urolithiasis: diagnostic yield for assessment of alternative diagnoses. Am J Roentgenol.

[18] Ronneberger O, Fischer P, Brox T. U-net: convolutional networks for biomedical image segmentation. In: International conference on medical image computing and computer-assisted intervention. Germany: Springer; 2015. p. 234–241.

[19] Imaging IT solutions that lead the way in customer satisfaction | Sectra Medical. https://medical.sectra.com/. Accessed 5 Dec 2020.

[20] Iakovidis DK, Goudas T, Smailis C, Maglogiannis I (2014). Ratsnake: a versatile image annotation tool with application to computer-aided diagnosis. Sci World J.

[21] Ioffe S and Szegedy C. Batch normalization: accelerating deep network training by reducing internal covariate shift. ArXiv150203167 Cs. 2015. Available: http://arxiv.org/abs/1502.03167. Accessed 11 Dec 2020.

[22] Liu M, Wu W, Gu Z, Yu Z, Qi F, Li Y (2018). Deep learning based on batch normalization for P300 signal detection. Neurocomputing.

[23] Shrestha A and Mahmood A. Optimizing Deep neural network architecture with enhanced genetic algorithm. In: 2019 18th IEEE International Conference on Machine Learning and Applications (ICMLA). USA: IEEE; 2019. p. 1365–1370.

[24] Xiao X, Yan M, Basodi S, Ji C and Pan Y. Efficient Hyperparameter optimization in deep learning using a variable length genetic algorithm. ArXiv Prepr. ArXiv200612703. 2020.

[25] Buslaev A, Iglovikov VI, Khvedchenya E, Parinov A, Druzhinin M, Kalinin AA (2020). Albumentations: fast and flexible image augmentations. Information.

[26] Zou KH (2004). Statistical validation of image segmentation quality based on a spatial overlap index. Acad Radiol.

[27] Koo TK, Li MY (2016). Cracking the code: providing insight into the fundamentals of research and evidence-based practice a guideline of selecting and reporting intraclass correlation coefficients for reliability research. J Chiropr Med.

[28] Sharma K (2017). Automatic segmentation of kidneys using deep learning for total kidney volume quantification in autosomal dominant polycystic kidney disease. Sci Rep.

[29] Talhar SS, Waghmare JE, Paul L, Kale S, Shende MR (2017). Computed tomographic estimation of relationship between renal volume and body weight of an individual. J Clin Diagn Res JCDR.

[30] Sah R, Bhattarai M, Pradhan BL, Shrestha SL, Lohani B, Bhatta R (2020). Computed tomographic assessment of renal volume and its associative factors among adults. J Nepal Health Res Counc.

[31] Beckers R, Kwade Z, Zanca F (2021). The EU medical device regulation: implications for artificial intelligence-based medical device software in medical physics. Phys Med.

[32] Jungmann F (2020). Towards data-driven medical imaging using natural language processing in patients with suspected urolithiasis. Int J Med Inf.

[33] Hosny A, Parmar C, Quackenbush J, Schwartz LH, Aerts HJWL (2018). Artificial intelligence in radiology. Nat Rev Cancer.

